# 3D Mueller matrix mapping of layered distributions of depolarisation degree for analysis of prostate adenoma and carcinoma diffuse tissues

**DOI:** 10.1038/s41598-021-83986-4

**Published:** 2021-03-04

**Authors:** Volodymyr A. Ushenko, Benjamin T. Hogan, Alexander Dubolazov, Gennadii Piavchenko, Sergey L. Kuznetsov, Alexander G. Ushenko, Yuriy O. Ushenko, Mykhailo Gorsky, Alexander Bykov, Igor Meglinski

**Affiliations:** 1 Optics and Publishing Department, Chernivtsi National University, 2 Kotsiubynskyi Str., Chernivtsi, 58012 Ukraine; 2 Optoelectronics and Measurement Techniques Laboratory, University of Oulu, 90014 Oulu, Finland; 3 Institute of Clinical Medicine N.V. Sklifosovsky, I.M. Sechenov First Moscow State Medical University, Moscow, Russia 129090; 4 Institute of Engineering Physics for Biomedicine (PhysBio), National Research Nuclear University (MEPhI), Moscow, Russia 115409; 5 College of Engineering and Physical Sciences, Aston University, Birmingham, B4 7ET UK

**Keywords:** Nanoscale biophysics, Polarization microscopy, Biophysical methods, High-throughput screening, Microscopy, Imaging, 3-D reconstruction, Circular dichroism, Structure determination, Cancer imaging, Cancer screening, Cancer imaging, Statistics, Characterization and analytical techniques, Imaging techniques, Applied optics, Optical physics, Nanophotonics and plasmonics, Nonlinear optics, Imaging and sensing, Optical techniques, Circular dichroism, Other photonics, Biophotonics, Biophysics, Nanoscience and technology, Optics and photonics, Applied physics, Biological physics, Techniques and instrumentation, Physics, Optical physics, Nanophotonics and plasmonics

## Abstract

Prostate cancer is the second most common cancer globally in men, and in some countries is now the most diagnosed form of cancer. It is necessary to differentiate between benign and malignant prostate conditions to give accurate diagnoses. We aim to demonstrate the use of a 3D Mueller matrix method to allow quick and easy clinical differentiation between prostate adenoma and carcinoma tissues with different grades and Gleason scores. Histological sections of benign and malignant prostate tumours, obtained by radical prostatectomy, were investigated. We map the degree of depolarisation in the different prostate tumour tissues using a Mueller matrix polarimeter set-up, based on the superposition of a reference laser beam with the interference pattern of the sample in the image plane. The depolarisation distributions can be directly related to the morphology of the biological tissues. The dependences of the magnitude of the 1st to 4th order statistical moments of the depolarisation distribution are determined, which characterise the distributions of the depolarisation values. To determine the diagnostic potential of the method three groups of histological sections of prostate tumour biopsies were formed. The first group contained 36 adenoma tissue samples, while the second contained 36 carcinoma tissue samples of a high grade (grade 4: poorly differentiated—4 + 4 Gleason score), and the third group contained 36 carcinoma tissue samples of a low grade (grade 1: moderately differentiated—3 + 3 Gleason score). Using the calculated values of the statistical moments, tumour tissues are categorised as either adenoma or carcinoma. A high level (> 90%) accuracy of differentiation between adenoma and carcinoma samples was achieved for each group. Differentiation between the high-grade and low-grade carcinoma samples was achieved with an accuracy of 87.5%. The results demonstrate that Mueller matrix mapping of the depolarisation distribution of prostate tumour tissues can accurately differentiate between adenoma and carcinoma, and between different grades of carcinoma. This represents a first step towards the implementation of 3D Mueller matrix mapping for clinical analysis and diagnosis of prostate tumours.

## Introduction

Prostate cancer is the second most common cancer globally in men, and in some countries is now the most diagnosed form of cancer^1,2^. Early diagnosis, intervention, and management can give significantly improved patient outcomes^3,4^. It is necessary to differentiate between benign (adenoma) and malignant (carcinoma) prostate tissue types, and then to further differentiate between grades of tumour tissues^4^. Optical analysis of tissue types offers distinct advantages, being non-destructive, requiring limited sample preparation, and being cheap and fast^5–10^. Analysis of the polarisation properties- known as polarimetric diagnostics- of different tissues holds great promise. Polarimetric diagnostics of optically anisotropic biological tissues are undergoing active development in biomedical optics^11–15^. Several key strands of investigation have emerged, including: scattering matrices^5,16,17^; Mueller matrix polarimetry^10,18–23^; polar decomposition of Mueller matrices^24,25^; and two-dimensional Mueller matrix mapping^26–29^ using various approximations^6–9^.

Mueller matrix mapping is particularly promising. Experimental and analytical results obtained using Mueller matrices were traditionally represented as 1D angular dependences (indicatrices) of matrix elements^10,13,24^. The next step in the development of the Mueller matrix polarimetry techniques was the use of digital cameras to obtain of 2D Mueller matrix images^21–23^, i.e. 2D distributions of the elements of the Mueller matrices. For the 2D distributions, the information obtained about the optical properties of the object being diagnosed is integrally averaged over the entire volume of the sample. However, most biological tissues have complex, spatially heterogeneous, structures and a pronounced depolarising ability. Therefore, the further development of new Mueller matrix polarimetry techniques which can provide 3D information is a critical challenge. This work focuses on the development and experimental testing of a new method of 3D Mueller matrix mapping of the anisotropic polarisation properties. Digital holographic reconstruction of 3D layered distributions of the degree of depolarisation is used for express diagnosis and differentiation of diffuse samples of histological sections of prostate tumour biopsy obtained by radical prostatectomy.

## Methods and theory

### Biological samples

**Figure 1:**
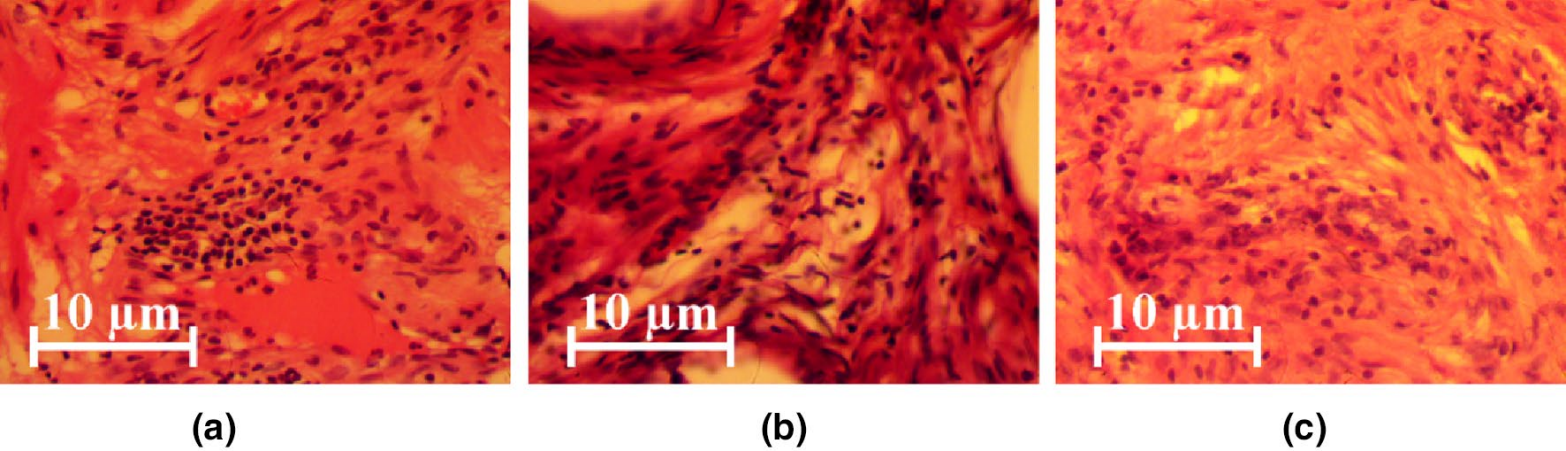
Representative real colour microscopic images (magnification ×50, biological microscope Ulab XY-B2T LED, digital camera UCMOSS08000KPB) of: (**a**) a prostate adenoma sample, (**b**) a moderately differentiated (3 + 3) prostate carcinoma sample, and (**c**) a poorly differentiated (4 + 4) prostate carcinoma sample.

For determination of the type of tumour, native samples of histological sections of the examined prostate tissue were made. This study was conducted in accordance with the principles of the Declaration of Helsinki, and in compliance with the International Conference on Harmonisation-Good Clinical Practice and local regulatory requirements. Ethical approval was obtained from the Ethics Committee of the Bureau of Forensic Medicine of the Chernivtsi National University and the Bukovinian State Medical University (Chernivtsi, Ukraine), and written informed consent was obtained from all subjects prior to study initiation. The type of prostate tumour was determined by an independent assessment of stained histological samples (Fig. 1). The histological analysis was conducted by the following procedure:

1. Fixation of prostate tissue with formalin (40% formaldehyde aqueous solution);
2. Washing of samples in running water for 24 h;
3. Dehydration with alcohols with increasing concentration (70–100%) within 48 h;
4. Fixing the material in a xylene-paraffin mixture for 1–2 h, at a temperature of 52°–56°, and cutting out a block with a sample enclosed in it;
5. Making histological sections on a standard freezing microtome;
6. Staining of histological sections with haematoxylin and eosin (Fig. 1 shows real colour microscopic images);
7. Microscopic examination of images of the obtained samples with differentiation of their structure by grades and determination of the position of the prostate tumor sample according to the Gleason Pattern scale.

Three representative groups of histological biopsy sections, obtained from radical prostatectomy, were formed:

- Group 1 consisted of $n={36}$ adenoma samples;
- Group 2 consisted of $n={36}$ moderately differentiated (3 + 3 on Gleason’s pattern scale) carcinoma samples;
- Group 3 consisted of $n={36}$ poorly differentiated (4 + 4 on Gleason’s Pattern scale) carcinoma samples.

Table 1 presents the optical and geometric parameters of the samples of native histological sections of prostate tumour biopsies from each of the groups. The geometric thickness of the histological sections was determined according to the standard values of the freezing microtome scale. The extinction coefficient was determined by measuring the attenuation of the illuminating beam intensity as it passed through the sample. This was achieved using an integral light-scattering sphere following a well-established method^30, 31^. The measurement of the integral degree of depolarisation ($\Lambda$) was performed using a standard Mueller matrix polarimeter in accordance with similar measurements previously underatken^23^.

**Table 1:** Optical and geometric parameters of histological sections of prostate tissue.

Parameter	Group 1	Group 2	Group 3
Geometric thickness $h,\,\upmu {\text{m}}$	40 ± 0.95	40 ± 0.95	40 ± 0.95
Attenuation (extinction) coefficient $\tau ,\;{\text{cm}}^{ - 1}$	2.01 ± 0.041	1.96 ± 0.037	1.99 ± 0.039
Depolarisation degree $\Lambda , \%$	81 ± 0.78	83 ± 0.81	79 ± 0.73

By comparing the optical and geometric parameters of the different histological section groups, we observe that the depolarisation degree is high for all samples. Additionally, there is little difference in the depolarisation values for the different groups. As discussed below, this significantly limits the diagnostic potential of traditional 2D Mueller matrix polarimetry.

### Theoretical background

The diagnostic efficiency of detection of oncological conditions by applying the technique of 2D Mueller matrix polarimetry to the polycrystalline component of optically thin (non-depolarising) histological sections of biological tissues of various human organs has previously been demonstrated^32–35^. These previous studies are devoted to the search for, and subsequent diagnostic application of, a set of diagnostically relevant relationships between Mueller matrix images ($F_{{{\text{ik}}}} \left( {\text{x,y}} \right)$) and maps of linear and circular birefringence ($LB\left( {{\text{x.y}}} \right)$, $CB\left( {{\text{x,y}}} \right)$) and dichroism ($LD\left( {{\text{x.y}}} \right)$, $CD\left( {{\text{x.y}}} \right)$):

1 $$F\left( {{\text{x}} \cdot {\text{y}}} \right) = \left\| {\begin{array}{*{20}c} {F_{11} } & {F_{12} } & {F_{13} } & {F_{14} } \\ {F_{21} } & {F_{22} } & {F_{23} } & {F_{24} } \\ {F_{31} } & {F_{32} } & {F_{33} } & {F_{34} } \\ {F_{41} } & {F_{42} } & {F_{43} } & {F_{44} } \\ \end{array} } \right\|\left( {{\text{x}} \cdot {\text{y}}} \right) \Leftrightarrow \left( {\begin{array}{*{20}c} {LB} \\ {CB} \\ {LD} \\ {CD} \\ \end{array} } \right)\left( {{\text{x}} \cdot {\text{y}}} \right).$$

As a general rule, samples of biological tissues of human organs are diffuse and thus strongly depolarize optical radiation (as is the case with our prostate tissue samples). This significantly limits the potential for differential diagnostics by traditional 2D Mueller matrix polarimetry. In this situation, almost all off-diagonal ($F_{ik,i \ne k}$) elements of the Mueller matrix of the diffuse biological layer are significantly reduced^5,17,21^. However, the diagonal elements ($F_{ik,i = k}$) are still diagnostically relevant. The superposition of the diagonal elements determines the overall value of depolarisation $\Lambda$ for the optical radiation^11,14,15^.

2 $$\left\{ {\begin{array}{*{20}c} {F_{ik,i \ne k} \to 0;} \\ {F_{ik,i = k} \ne 0;} \\ \end{array} } \right.$$

3 $$F = \left\| {\begin{array}{*{20}c} {F_{11} } & 0 & 0 & 0 \\ 0 & {F_{22} } & 0 & 0 \\ 0 & 0 & {F_{33} } & 0 \\ 0 & 0 & 0 & {F_{44} } \\ \end{array} } \right\| \Leftrightarrow \Lambda ;$$

4 $$\Lambda = 1 - \frac{1}{3}\left( {F_{22} + F_{33} + F_{44} } \right).$$

The value of the parameter $\Lambda$ is an integral equivalent of the overall optical properties of the object under investigation.

Analysing (4), one can identify the following range of variation of the parameter $\Lambda$:

- for an optically homogeneous isotropic layer $(F_{{22;33;44}} \to 1)$, and thus $\Lambda \to 0$;
- for an ideal diffuser $(F_{{22;33;44}} \to 0)$, and thus $\Lambda \to 1$.

In all other cases, the value of the integral depolarisation parameter is determined by a combination of two components. Firstly, the ‘local’ component resulting from the formation of an orthogonal component of the laser radiation amplitude (i.e. a change in the initial state of polarisation) due to the optical anisotropy of the biological layer. We will call this the “A-mechanism”. Secondly, the ‘diffuse’ component resulting from the statistical averaging of the polarisation state due to the superposition of laser waves, scattered in the volume of the biological layer, with different states of polarisation. We will call this the “B-mechanism”.

For optically thin ($\tau \le 0.01$), singly-scattering, optically anisotropic biological layers, the A-mechanism is dominant. The influence of the optical anisotropy (linear and circular birefringence and dichroism) is seen through a slight decrease in the values of the diagonal elements ${F}_{22;33;44}<1$ of the Mueller matrix^23^. Therefore, in comparison with an optically isotropic layer, there is an increase in the value of depolarisation ($\Lambda >0$). The specific value of this parameter is interrelated with the specificity of the polycrystalline structure of biological tissues^10,32,35^.

As the optical thickness increases ($\tau >0.01$), the multiplicity of the light scattering (i.e. the number of scattering events each photon experiences on average) in the volume of the histological sections increases correspondingly. The value of the integral depolarisation parameter $\Lambda$ is determined by the superposition of the effects of the A- and B-mechanisms. For small values of the attenuation coefficient ($\tau \le 0.5$), the contribution of the two optical mechanisms is comparable. Averaging the polarisation states of the scattered (differently polarized) wavefronts leads to a further increase in the degree of integral depolarisation. There is hence a decrease in the dependence of integral depolarisation on the specific morphological structure of biological tissue.

For diffuse (optically thick, $\tau >1.5$) biological layers, the polarising effects of the individual features of the polycrystalline structure are effectively smoothed by the B-mechanism. Therefore, the effectiveness of differential 2D diagnostics is extremely low or unsatisfactory in this limit. The 2D polarisation diagnostics of frozen prostate tissue samples is limited by the impossibility of obtaining optically thin ($h \sim 10{-}15\,\upmu{\text{m}}$) histological sections with geometric consistency between samples. To obtain consistent samples, a geometric thickness $h \sim 40\,\upmu{\text{m}}$ was required. Hence, the B-mechanism is dominant for all samples, with the diffuse scattering resulting in both high and comparable values of the integral depolarisation for all the types of prostate tissues investigated (Table 1).

### Experimental setup

**Figure 2:**
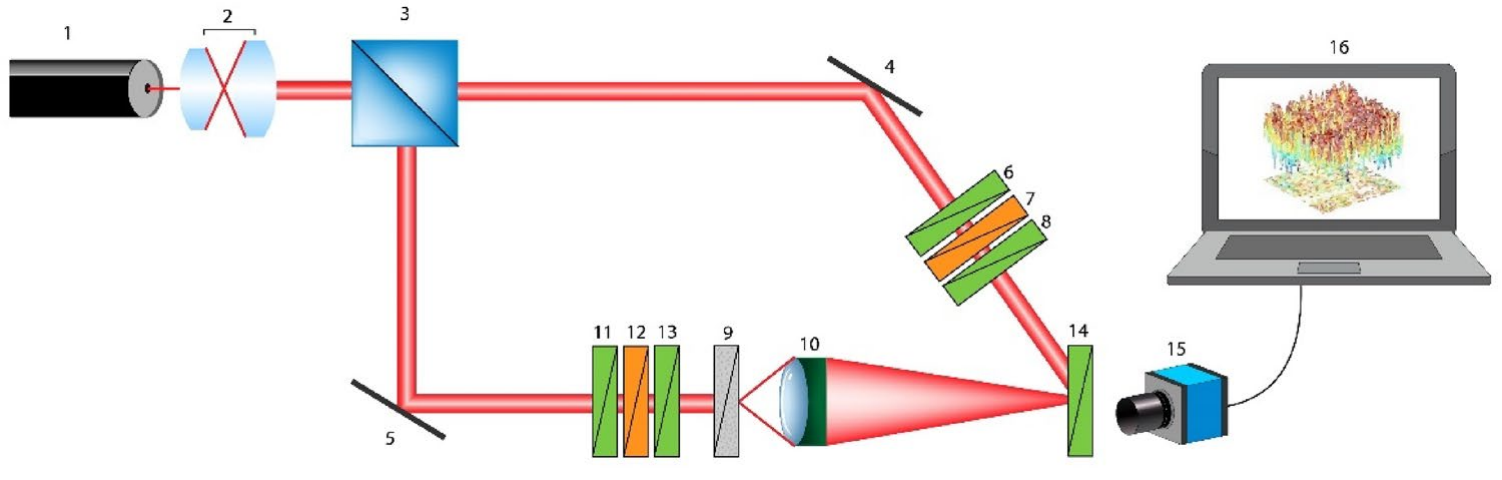
Optical scheme of 3D Mueller matrix polarimetry. Here: 1—laser; 2—collimator; 3—beam splitter; 4,5—reflecting mirrors; 6,8,11,13,14—polarisers; 7,12—quarter-wave phase plates; 9—object; 10—strain-free polarisation objective; 15—digital camera; 16—processing unit.

The method of 3D Mueller matrix mapping is based on the use of a reference wave of laser radiation. The reference wave is superimposed on the polarisation-inhomogeneous image of the biological layer using an optical interferometry scheme (Fig. 2).

Experimentally herein, the parallel ($\oslash =2\times 1{0}^{3}\,\upmu{\text{m}}$) beam of a He–Ne ($\lambda =0.6328\,\upmu{\text{m}}$) laser (1, see Fig. 2) (Edmund Optics Lumentum High Performance Helium–Neon Laser, 5mW), formed by passing through a collimator (2, see Fig. 2), is divided by a 50/50 beam splitter (3, see Fig. 2) into separate illuminating and reference beams. The illuminating beam is directed by a mirrors (5, see Fig. 2) through a polarising (11–13, see Fig. 2) consisting of two linear polarisers (Edmund Optics TechSpec High Contrast Glass Linear Polarizer) (11 and 13, see Fig. 2) and a quarter wave plate (Astropribor Achromatic Quarterwave Plate APAW-20) (12, see Fig. 2). The polarised illuminating beam then passes through the sample (9, see Fig. 2). A polarisation-inhomogeneous image of the object is projected through a strain-free microscopic objective(10, see Fig. 2) (Nikon CFI Achromat P, 4x) into the imaging plane of the digital camera (15, see Fig. 2) (The Imaging Source DMK 41AU02.AS, monochrome 1/2" CCD, Sony ICX205AL). The reference beam is directed by a mirror (14, see Fig. 2) through an equivalent polarising filter (6–8, see Fig. 2) (optical components are the same as (11–13, see Fig. 2)) into the plane of the polarisation-inhomogeneous image of the object. The angle between the illuminating and reference beams was selected as $\Psi =4^{\circ}$. As a result, an interference pattern is formed, the period of which is $10\,\upmu{\text{m}}$. According to the Nyquist–Shannon sampling theorem, this periodicity ensures reliable recording of a single fringe by 5 pixels of the digital camera (15, see Fig. 2).

The error in setting the transmission axes of the polarisers^8,11,13,14,36^ was $\Delta \gamma = 0.2^\circ$. The phase shift error of the quarter-wave plates^13,37^ was $\Delta \delta ={0.5}^{\circ}$. The error in setting the fast axis of the quarter-wave plates was $\Delta \varphi =0.2^\circ$. The resulting error in the linear polarisation states did not exceed 0.0021, and that in the circular polarisation states did not exceed 0.0056^38^.

Before carrying out measurements on histological sections of the prostate, metrological certification of the experimental setup was conducted using model objects (air, linear polariser, quarter- and half-wave plates). From 50 measurements for each model object, the errors in the determination of the diagonal elements of the Mueller matrix were determined: for ${F}_{22;33}$- 0.25% and for ${F}_{44}$- 0.5%.

### Determination of the layered depolarisation distributions

When obtaining the polarisation interference images, one can determine the Mueller matrix elements by the following procedure:

1. Forming six distinct polarisation states of both the illuminating and reference beams: four linear polarisations at angles of 0°, 90°, 45°, and 135°, and two circular polarisations—right ($\otimes$) and left ($\oplus$) circular polarisation respectively.
2. Collection of six images for each partial interference pattern passed through the polariser-analyser [14] with sequential orientation of its transmission axis at angles $$\begin{array}{cc}{\Omega }_{0}={0}^{\circ};& {\Omega }_{90}=9{0}^{\circ}\end{array}$$. We then have a series of twelve digital photo of the original field with the applied reference wave.
3. For each partial interference distribution, we perform a two-dimensional discrete Fourier transform (${\text{DFT}}\left(\upsilon ,\nu \right))$ on the image. The two-dimensional ${\text{DFT}}\left(\upsilon ,\nu \right)$ of a two-dimensional array ${I}_{0;90}\left(\text{x,y}\right)$ (i.e. the image) f(x,y)is a function of two discrete variables coordinates $\left(\text{x,y}\right)$ the ${\text{DFT}}\left(\upsilon ,\nu \right)$ function is defined by by^39^:
  5 $$DFT_{{\Omega = 0^{\circ};90^{\circ}}}^{{0^{\circ};90^{\circ};45^{\circ};135^{\circ}; \otimes ; \oplus }} \left( {\upsilon ,\nu } \right) = \frac{1}{M \times N}\mathop \sum \limits_{x = 0}^{M - 1} \mathop \sum \limits_{y = 0}^{N - 1} I_{{\Omega = 0^{\circ} ;90^{\circ}}} \left( {x,y} \right)\exp \left[ { - i2\pi \left( {\frac{x \times \upsilon }{M} + \frac{y \times \nu }{N}} \right)} \right]$$
  where ${I}_{0^{\circ};90^{\circ}}\left(\text{x,y}\right)={A}_{0^{\circ};90^{\circ}}\left(\text{x,y}\right){{A}_{0^{\circ};90^{\circ}}}^{*}\left(\text{x,y}\right)$ are the coordinate distributions of the intensity of the interference pattern (for each polarisation state of the illuminating and reference beams) filtered by the analyser with the orientation of its transmission axis at ${\Omega }_{0^{\circ}}={0}^{\circ}$ and ${\Omega }_{90^{\circ}}=9{0}^{\circ}$; ${A}_{0^{\circ};90^{\circ}}\left(\text{x,y}\right)$ are the orthogonal projections of the complex amplitudes; $*$ denotes the complex conjugation operation; $\left(\upsilon ,\nu \right)$ are the spatial frequencies in the x and y directions respectively; and $\left(\text{M,N}\right)$ are the number of pixels of the CCD camera in the x and y directions respectively, such that $0\le \text{x,}\upsilon \le M$ and $0\le \text{y,}\nu \le N$.
4. The results of this transformation should contain three peaks, one central (main) peak and two additional side peaks. The Fourier transform acts like a low-pass filter. It removes interference fringes, which enables extraction of the complex representation of the real field due to the object. Also, since the extracted part has limited size, it acts like a low-pass filter for the object field too.
5. Either of the additional side peaks (in complex representation) can be used to create a new Fourier spectrum by first extracting the peak and then placing it into centre of a newly generated spectrum $DFT_{{\Omega = 0^{\circ};90^{\circ}}}^{{0^{\circ};90^{\circ};45^{\circ};135^{\circ}; \otimes ; \oplus }} \left( {\upsilon ,\nu } \right)$.
6. Applying a two-dimensional inverse discrete Fourier transform ${\left(DF{T}_{\Omega ={0}^{\circ};9{0}^{\circ}}^{{0}^{\circ};9{0}^{\circ};4{5}^{\circ};13{5}^{\circ};\otimes ;\oplus }\right)}^{-1}\left(x,y\right)$ on the obtained spectrum ${\text{DFT}}_{{\Omega = 0^{\circ};90^{\circ}}}^{{0^{\circ};90^{\circ};45^{\circ};135^{\circ}; \otimes ; \oplus }} \left( {\upupsilon ,\upnu } \right)$, one gets:
  6 $$\left( {DFT_{{\Omega = 0^{\circ};90^{\circ}}}^{{0^{\circ};90^{\circ};45^{\circ};135^{\circ}; \otimes ; \oplus }} } \right)^{ - 1} \left( {x,y} \right) = \frac{1}{M \times N}\mathop \sum \limits_{x = 0}^{M - 1} \mathop \sum \limits_{y = 0}^{N - 1} DFT_{{\Omega = 0^{\circ};90^{\circ}}}^{{0^{\circ};90^{\circ};45^{\circ};135^{\circ}; \otimes ; \oplus }} \left( {\upsilon ,\nu } \right)\exp \left[ { - i2\pi \left( {\frac{x \times \upsilon }{M} + \frac{y \times \nu }{N}} \right)} \right].$$
  Here, $$\left( {DFT_{{\Omega = 0^{\circ};90^{\circ}}}^{{0^{\circ};90^{\circ};45^{\circ};135^{\circ}; \otimes ; \oplus }} } \right)^{ - 1} \left( {x,y} \right) \equiv A_{{\Omega = 0^{\circ};90^{\circ}}}^{{0^{\circ};90^{\circ};45^{\circ};135^{\circ}; \otimes ; \oplus }} \left( {x,y} \right).$$
7. One subsequently obtains (for each polarisation state) a distribution of complex amplitudes:
  7 $$\left\{ {\begin{array}{*{20}c} {\Omega_{0^{\circ}} \to \left| {A_{0^{\circ}} } \right|} \\ {\Omega_{90^{\circ}} \to \left| {A_{90^{\circ}} } \right|\exp \left( {i\left( {\delta_{90^{\circ}} - \delta_{0^{\circ}} } \right)} \right)} \\ \end{array} } \right.$$
  in different phase planes (which are subplanes of the image). The phase planes are defined by:
  8 $$\theta_{k} = \left( {\delta_{90^{\circ}} - \delta_{0^{\circ}} } \right) = \frac{2\pi }{\lambda }\Delta nz\begin{array}{*{20}c}; & {0 \le z \le h} \\ \end{array}$$
  where $\Delta n$ is the birefringence; $\lambda$ is the wavelength; and $h$ is the sample thickness of the object field, separated by an arbitrary step of $\Delta \theta$.
8. In each phase plane $\theta_{k}$, the corresponding sets of parameters of the Stokes vector of the object field of the biological layer are calculated:
  9 $$\begin{aligned} VS_{1}^{0^{\circ};90^{\circ};45^{\circ};135^{\circ}; \otimes ; \oplus } & = \left( {\left| {A_{0^{\circ}} } \right|^{2} + \left| {A_{90^{\circ}} } \right|^{2} } \right)^{0^{\circ};90^{\circ};45^{\circ};135^{\circ}; \otimes ; \oplus } ; \\ VS_{2}^{0^{\circ};90^{\circ};45^{\circ};135^{\circ}; \otimes ; \oplus } & = \left( {\left| {A_{0^{\circ}} } \right|^{2} - \left| {A_{90^{\circ}} } \right|^{2} } \right)^{0^{\circ};90^{\circ};45^{\circ};135^{\circ}; \otimes ; \oplus } ; \\ VS_{3}^{0^{\circ};90^{\circ};45^{\circ};135^{\circ}; \otimes ; \oplus } & = \left( {2\left| {A_{0^{\circ}} } \right|^{2} \left| {A_{90^{\circ}} } \right|^{2} \cos \theta_{k} } \right)^{0^{\circ};90^{\circ};45^{\circ};135^{\circ}; \otimes ; \oplus } ; \\ VS_{4}^{0^{\circ};90^{\circ};45^{\circ};135^{\circ}; \otimes ; \oplus } & = \left( {2\left| {A_{0^{\circ}} } \right|^{2} \left| {A_{90^{\circ}} } \right|^{2} \sin \theta_{k} } \right)^{0^{\circ};90^{\circ};45^{\circ};135^{\circ}; \otimes ; \oplus } . \\ \end{aligned}$$

The obtained relations (9) form the basis of the method for determining layered distributions of the values of the Mueller matrix elements ${F}_{ik}\left(x,y,{\theta }_{k}\right)$. The elements are obtained from the following relationships:

1. For Stokes vectors of linearly polarized probe beams, $$V{S}^{0}\begin{array}{cc}\left({0}^{\circ}\right);& V{S}^{0}\left(9{0}^{\circ}\right)\end{array}$$:
  10 $$\left\{\begin{array}{c}\left[V{S}^{*}\left({0}^{\circ}\right)=\left\{F\right\}\left(\begin{array}{c}1\\ 1\\ 0\\ 0\end{array}\right)=\left(\begin{array}{c}{F}_{11}+{F}_{12}\\ {F}_{21}+{F}_{22}\\ {F}_{31}+{F}_{32}\\ {F}_{41}+{F}_{42}\end{array}\right)\right];\\ \left[V{S}^{*}\left(9{0}^{\circ}\right)=\left\{F\right\}\left(\begin{array}{c}1\\ -1\\ 0\\ 0\end{array}\right)=\left(\begin{array}{c}{F}_{11}-{F}_{12}\\ {F}_{21}-{F}_{22}\\ {F}_{31}-{F}_{32}\\ {F}_{41}-{F}_{42}\end{array}\right)\right]\end{array}\right\}\left({\theta }_{k}\right)\Rightarrow {F}_{ik}\left({\theta }_{k}\right)=\left\Vert \begin{array}{cc}{F}_{11}& {F}_{12}\\ {F}_{21}& {F}_{22}\\ {F}_{31}& {F}_{32}\\ {F}_{41}& {F}_{42}\end{array}\right\Vert \left({\theta }_{k}\right).$$
2. For Stokes vectors of linearly polarized probe beams $$V{S}^{0}\begin{array}{cc}\left(4{5}^{\circ}\right);& V{S}^{0}\left(13{5}^{\circ}\right)\end{array}$$:
  11 $$\left\{\begin{array}{c}\left[V{S}^{*}\left(4{5}^{\circ}\right)=\left\{F\right\}\left(\begin{array}{c}1\\ 0\\ 1\\ 0\end{array}\right)=\left(\begin{array}{c}{F}_{11}+{F}_{13}\\ {F}_{21}+{F}_{23}\\ {F}_{31}+{F}_{33}\\ {F}_{41}+{F}_{43}\end{array}\right)\right];\\ \left[V{S}^{*}\left(13{5}^{\circ}\right)=\left\{F\right\}\left(\begin{array}{c}1\\ 0\\ -1\\ 0\end{array}\right)=\left(\begin{array}{c}{F}_{11}-{F}_{13}\\ {F}_{21}-{F}_{23}\\ {F}_{31}-{F}_{33}\\ {F}_{41}-{F}_{43}\end{array}\right)\right]\end{array}\right\}\left({\theta }_{k}\right)\Rightarrow {F}_{ik}\left({\theta }_{k}\right)=\left\Vert \begin{array}{cc}{F}_{11}& {F}_{13}\\ {F}_{21}& {F}_{23}\\ {F}_{31}& {F}_{33}\\ {F}_{41}& {F}_{43}\end{array}\right\Vert \left({\theta }_{k}\right).$$
3. For Stokes vectors of right- and left-circularly polarized probe beams $$V{S}^{0}\begin{array}{cc}\left(\otimes \right);& V{S}^{0}\left(\oplus \right)\end{array}$$:
  12 $$\left\{\begin{array}{c}\left[V{S}^{*}\left(\otimes \right)=\left\{F\right\}\left(\begin{array}{c}1\\ 0\\ 0\\ 1\end{array}\right)=\left(\begin{array}{c}{F}_{11}+{F}_{14}\\ {F}_{21}+{F}_{24}\\ {F}_{31}+{F}_{34}\\ {F}_{41}+{F}_{44}\end{array}\right)\right];\\ \left[V{S}^{*}\left(\oplus \right)=\left\{F\right\}\left(\begin{array}{c}1\\ 0\\ 0\\ -1\end{array}\right)=\left(\begin{array}{c}{F}_{11}-{F}_{14}\\ {F}_{21}-{F}_{24}\\ {F}_{31}-{F}_{34}\\ {F}_{41}-{F}_{44}\end{array}\right)\right]\end{array}\right\}\left({\theta }_{k}\right)\Rightarrow {F}_{ik}\left({\theta }_{k}\right)=\left\Vert \begin{array}{cc}{F}_{11}& {F}_{14}\\ {F}_{21}& {F}_{24}\\ {F}_{31}& {F}_{34}\\ {F}_{41}& {F}_{44}\end{array}\right\Vert \left({\theta }_{k}\right).$$

From (9)–(12), working relations for determining the values of the elements of the Mueller matrix in the phase section ${\theta }_{k}$ are obtained:

13 $$\left\{F\left({\theta }_{j}\right)\right\}=0.5\left\Vert \begin{array}{cccc}\left(V{S}_{1}^{*}\left({0}^{\circ}\right)+V{S}_{1}^{*}\left(9{0}^{\circ}\right)\right)& \left(V{S}_{1}^{*}\left({0}^{\circ}\right)-V{S}_{1}^{*}\left(9{0}^{\circ}\right)\right)& \left(V{S}_{1}^{*}\left(4{5}^{\circ}\right)-V{S}_{1}^{*}\left(13{5}^{\circ}\right)\right)& \left(V{S}_{1}^{*}\left(\otimes \right)-V{S}_{1}^{*}\left(\oplus \right)\right)\\ \left(V{S}_{2}^{*}\left({0}^{\circ}\right)+V{S}_{2}^{*}\left(9{0}^{\circ}\right)\right)& \left(V{S}_{2}^{*}\left({0}^{\circ}\right)-V{S}_{2}^{*}\left(9{0}^{\circ}\right)\right)& \left(V{S}_{2}^{*}\left(4{5}^{\circ}\right)-V{S}_{2}^{*}\left(13{5}^{\circ}\right)\right)& \left(V{S}_{2}^{*}\left(\otimes \right)-V{S}_{2}^{*}\left(\oplus \right)\right)\\ \left(V{S}_{3}^{*}\left({0}^{\circ}\right)+V{S}_{3}^{*}\left(9{0}^{\circ}\right)\right)& \left(V{S}_{3}^{*}\left({0}^{\circ}\right)-V{S}_{3}^{*}\left(9{0}^{\circ}\right)\right)& \left(V{S}_{3}^{*}\left(4{5}^{\circ}\right)-V{S}_{3}^{*}\left(13{5}^{\circ}\right)\right)& \left(V{S}_{3}^{*}\left(\otimes \right)-V{S}_{3}^{*}\left(\oplus \right)\right)\\ \left(V{S}_{4}^{*}\left({0}^{\circ}\right)+V{S}_{4}^{*}\left(9{0}^{\circ}\right)\right)& \left(V{S}_{4}^{*}\left({0}^{\circ}\right)-V{S}_{4}^{*}\left(9{0}^{\circ}\right)\right)& \left(V{S}_{4}^{*}\left(4{5}^{\circ}\right)-V{S}_{4}^{*}\left(13{5}^{\circ}\right)\right)& \left(V{S}_{4}^{*}\left(\otimes \right)-V{S}_{4}^{*}\left(\oplus \right)\right)\end{array}\right\Vert \left({\theta }_{j}\right)$$

Finally, the distribution of the overall degree of depolarisation in each phase section is determined by the following relation:

14 $$\Lambda \left( {x,y,\theta_{k} } \right) = 1 - \frac{1}{3}\left\{ {\left[ {\left( {VS_{2}^{*} \left( {0^{\circ}} \right) - VS_{2}^{*} \left( {90^{\circ}} \right)} \right)} \right] + \left[ {\left( {VS_{3}^{*} \left( {45^{\circ}} \right) - VS_{3}^{*} \left( {135^{\circ}} \right)} \right)} \right] + \left[ {\left( {VS_{4}^{*} \left( \otimes \right) - VS_{4}^{*} \left( \oplus \right)} \right)} \right]} \right\}$$

### Statistical assessment of the polarisation distributions

The distributions of the values $\Lambda \left( {x,y,\theta_{k} } \right)$ can be quantitatively assessed by calculating the set of central statistical moments of the first to fourth orders $Z_{i = 1;2;3;4}$^14,32,34^ in each phase plane $\theta_{k}$:

15 $$Z_{1} = \frac{1}{K}\mathop \sum \limits_{j = 1}^{K} \Lambda_{j} ;$$

16 $$Z_{2} = \sqrt {\frac{1}{K}\mathop \sum \limits_{j = 1}^{K} (\Lambda - Z_{1} )_{j}^{2} } ;$$

17 $$Z_{3} = \frac{1}{{Z_{2}^{3} }}\frac{1}{K}\mathop \sum \limits_{j = 1}^{K} (\Lambda - Z_{1} )_{j}^{3} ;$$

18 $$Z_{4} = \frac{1}{{Z_{2}^{4} }}\frac{1}{K}\mathop \sum \limits_{j = 1}^{K} (\Lambda - Z_{1} )_{j}^{4} .$$

Here, $K$ is the total number of pixels of the CCD-camera. These parameters characterize the mean value ($Z_{1}$), dispersion ($Z_{2}$), skewness ($Z_{3}$) and kurtosis ($Z_{4}$) of the distributions $\Lambda \left( {x,y,\theta_{k} } \right)$.

To determine the statistical significance of a representative sampling of the number of samples by the cross-validation method^39^, the standard deviation $\sigma^{2}$ of each of the calculated values of the central statistical moments $Z_{{i = {1;2;3;4}}} \left( n \right)$ (15–18), which characterise the distribution of the degree of depolarisation $\Lambda$ (4), was determined. The specified number (36 for each group) of samples provided the level $\sigma^{2} \le 0.025$. This standard deviation corresponds to a confidence interval $p < {0}{\text{.05}}$, which demonstrates the statistical reliability of the 3D Mueller matrix mapping method for layered depolarisation maps within a representative sample.

### Diagnostic method

Our proposed optical procedure, used herein for the differential diagnosis of the prostate tissue samples, comprised the following steps:

1. We determined the optimal phase plane for further analysis and diagnostics. A "macro" step of discrete phase scanning was chosen—$\Delta \theta_{k}^{max} = 0.25\ rad$. Algorithmically, a series of layered coordinate distributions of the depolarisation degree ${\Lambda }\left( {x,y,\theta_{k} } \right)$ corresponding to each $\Delta \theta_{k}^{max} = 0.25\ rad$ was reconstructed.
2. For each phase section $\theta_{k}^{max}$ of the object field of scattered radiation, the set of central statistical moments of the first to fourth orders $Z_{i = 1;2;3;4}$, which characterize the distributions $\Lambda \left( {x,y,\theta_{k} } \right)$, was calculated.
3. The difference $\left( {\Delta Z_{i} } \right)_{k} = Z_{i} \left( {\theta_{j + 1}^{max} } \right) - Z_{i} \left( {\theta_{j}^{max} } \right)$ between the values of each of the statistical moments for the different phase planes was calculated.
4. The phase interval $\Delta \theta^{*} = \left( {\theta_{j + 1}^{max} - \theta_{j}^{max} } \right)$ was determined, within which the monotonic growth of the value $\Delta Z_{i} = Z_{i} \left( {\theta_{j + 1}^{max} } \right) - Z_{i} \left( {\theta_{j}^{max} } \right) \le 0$ stops.
5. Within the limits of $\Delta \theta^{*}$, a new series of values $\Delta Z_{i} = Z_{i} \left( {\theta_{q + 1}^{min} } \right) - Z_{i} \left( {\theta_{q}^{min} } \right)$ was calculated with a discrete phase scanning “micro” step $\Delta \theta_{q}^{min} = 0.05\ rad$.
6. The optimal phase plane $\theta^{*}$ was determined, in which $\Delta Z_{i} \left( {\theta^{*} } \right) = max$. The specified analytical procedure for one sample of prostate tissue took less than 7 min. A more accurate phase scanning algorithm can be chosen, for example with a step of 0.01 rad. In this case the processing time increased to 11 min.
7. In the plane $\theta^{*}$, the mean $\Delta \overline{Z}_{i = 1;2;3;4}^{*}$ and standard deviations $\sigma \left( {\Delta Z_{i}^{*} } \right)$ were determined within the representative samplings of histological sections from group 1, group 2 and group 3.
8. To differentiate benign and malignant tumours, for each of the statistical moments $Z_{i = 1;2;3;4}$, the sensitivity ($Se_{12} = \frac{{a_{12} }}{{a_{12} + b_{12} }}100\%$; $Se_{13} = \frac{{a_{13} }}{{a_{13} + b_{13} }}100\%$), specificity ($Sp_{12} = \frac{{c_{12} }}{{c_{12} + d_{12} }}100\%$; $Sp_{13} = \frac{{c_{13} }}{{c_{13} + d_{13} }}100\%$) and balanced accuracy ($Ac_{12} = \frac{{Se_{12} + Sp_{12} }}{2}$; $Ac_{13} = \frac{{Se_{13} + Sp_{13} }}{2}$) were calculated. Here, $a_{12}$ ($a_{13}$) and $b_{12}$ ($b_{13}$) are the number of correct and incorrect diagnoses within group 2 (group 3); and $c_{12}$ ($c_{13}$) and $d_{12}$ ($d_{13}$) are the same within group 1.
9. Similarly, to differentiate the grade of cancer, the sensitivity ($Se_{23} = \frac{{a_{23} }}{{a_{23} + b_{23} }}100\%$), specificity ($Sp_{23} = \frac{{c_{23} }}{{c_{23} + d_{23} }}100\%$) and balanced accuracy ($Ac_{23} = \frac{{Se_{23} + Sp_{23} }}{2}$) were calculated for each of the statistical moments $Z_{i = 1;2;3;4}$. Here, $a_{23}$ and $b_{23}$ the number of correct and incorrect diagnoses within group 3; while $c_{23}$ and $d_{23}$ are the same within group 2.

The position of the optimal phase plane $\theta^{ * }$, determined by the indicated methods for each of the three groups separately, coincided with an accuracy of 0.02 rad. The operating characteristics of the method^40^ (sensitivity $Se$, specificity $Sp$, accuracy $Ac$) remained practically unchanged.

## Results

**Figure 3:**
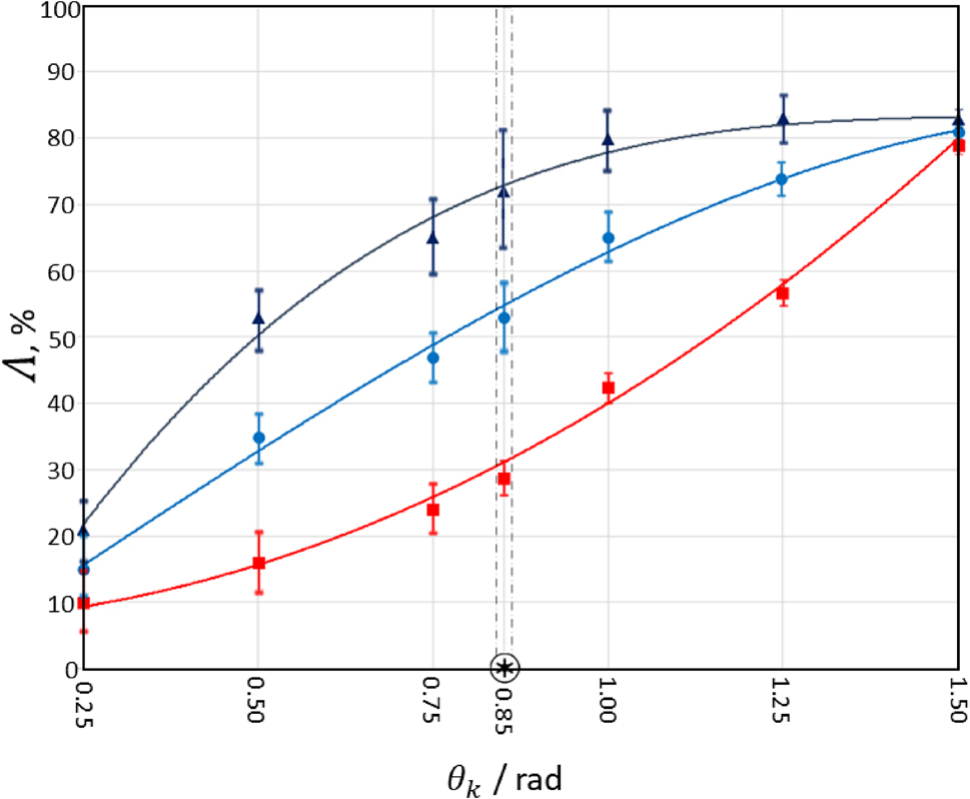
Dynamics of layered changes in the magnitude of the degree of depolarisation $\Lambda \left( {\theta_{k} } \right)$ by samples of histological sections of adenoma (dark blue curve), well differentiated (blue curve) and poorly differentiated (red curve) prostate carcinoma.

Figure 3 shows the phase dependences of the integral magnitude (averaged over all the pixels of the CCD-camera) of the degree of depolarisation $\Lambda \left( {\theta_{k} } \right)$ of laser radiation passing through prostate tissues from each of the three groups defined previously. The degree of depolarisation was experimentally determined using the 3D Mueller matrix mapping method described above.

Analysis of the data obtained (see Fig. 3) revealed that, despite the overall depolarisation through the whole samples being more or less the same (Table 1), there are significant differences observed within the volume of the tissue samples. The overall ranges of $\Lambda \left( {\theta_{k} } \right)$ by histological biopsy sections of all samples span $\left( {15\%{-}19\% } \right) for \left( {\theta_{k} = 0.25\,rad} \right) \le \Lambda \le \left( {78\%{-}81\% } \right) for \left( {\theta_{k} = 1.5\,{\text{rad}}} \right)$. The rate at which the dependencies $\Lambda \left( {\theta_{k} } \right)$ change is very different though. The most rapidly increasing depolarising ability is seen for samples of histological sections of adenoma biopsy (see Fig. 3, dark curve), while the least rapid is for samples of native sections of grade 4 carcinoma (see Fig. 3, red curve), with grade 1 carcinoma (see Fig. 3, blue line).

For small values of the phase ($\theta_{k} \le 0.5\,rad$), the dominant contribution to the depolarisation formation scenario is the “A—mechanism”. In general, soft biological tissues (including the prostate) have a relatively low level of optical anisotropy. The magnitude of the circular and linear birefringence $\Delta n$ does not exceed $1.5 \times 10^{ - 3}$^5–8^. Therefore, for small values of the phase, the integral depolarisation is low for histological sections of all types of prostate tumours. With increasing $\theta_{k}$, the contribution of the “B-mechanism” to the depolarising ability of the prostate samples increases. The contribution of the “B-mechanism” characterizes the measure of diffraction expansion of partial waves on optical inhomogeneities of biological tissue. The most pronounced effect of this mechanism manifests on small-scale (well differentiated) structures of the prostate tissue. Therefore, the most rapidly growing value of $\Lambda \left( {\theta_{k} } \right)$ is found for samples of histological sections of prostate adenoma (see Fig. 3, dark curve), the morphological structure of which is at the smallest scale. Conversely, the values of $\Lambda \left( {\theta_{k} } \right)$ for histological sections of biopsy of poorly differentiated prostate carcinoma increase most slowly (see Fig. 3, red curve).

For the largest values of phase shifts ($\theta_{k} \ge 1.5\,rad$), the diffraction “B—mechanism”, which characterizes the multiple superposition of laser waves scattered in the volume of the biological layer, becomes dominant. The degree of depolarisation then reaches a maximum level for all types of samples. The maximum differences between the values of integral depolarisation by samples of histological sections of biopsy of prostate tumours are realized for a certain “intermediate” range of phase shifts ($0.75\,rad < \Delta \theta^{*} < 1.05\,rad$). The maximum difference is found at $\theta^{*} = 0.85\,rad$, so we choose this value for further investigations.

**Figure 4:**
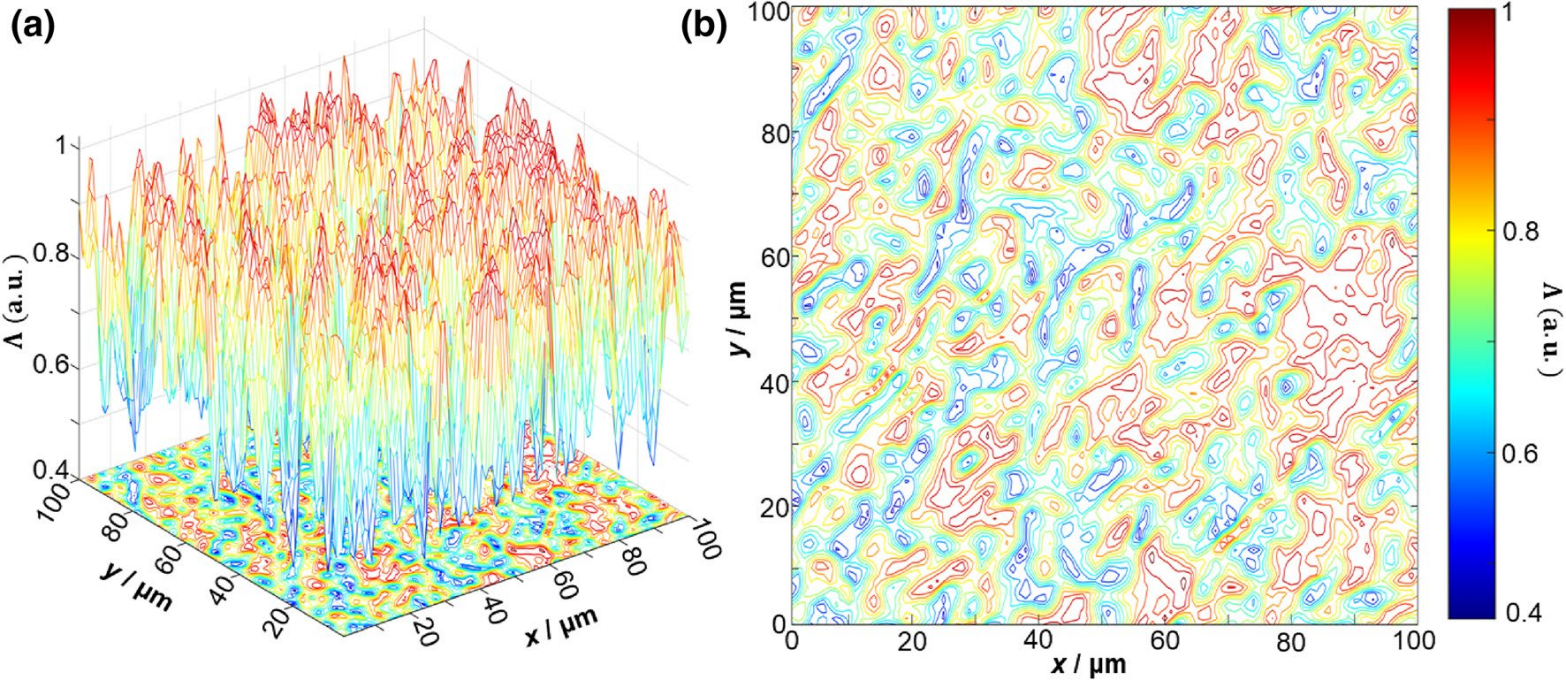
3D surfaces (**a**) and 2D contours (**b**) of depolarisation maps $\Lambda \left( {x,y,\theta_{{\text{k}}} = 0.85\,rad} \right)$ of a sample of a native histological section of adenoma biopsy during radical prostatectomy.

**Figure 5:**
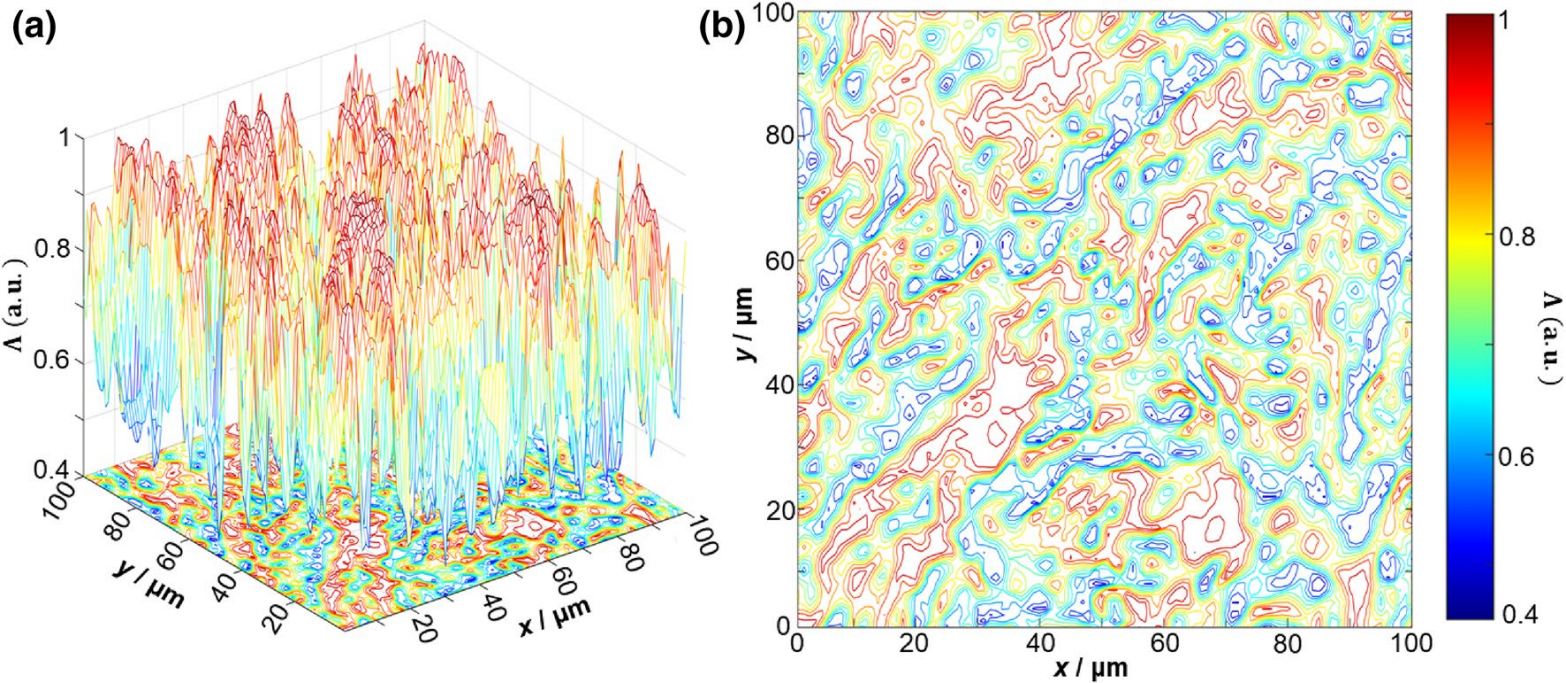
3D surfaces (**a**) and 2D contours (**b**) of depolarisation maps $\Lambda \left( {x,y,\theta_{k} = 0.85\,rad} \right)$ of a sample of a native histological biopsy section of a moderately differentiated (3 + 3 Gleason score) carcinoma during radical prostatectomy.

**Figure 6:**
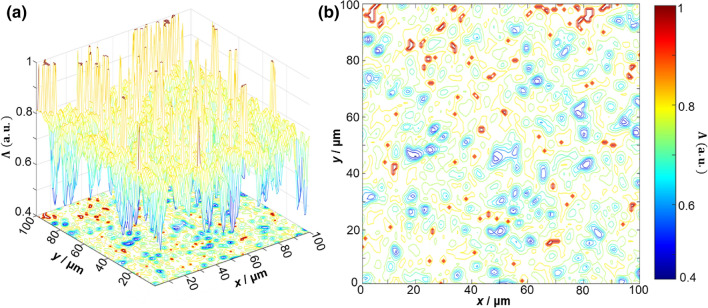
3D surfaces (**a**) and 2D contours (**b**) of depolarisation maps $\Lambda \left( {x,y,\theta_{{\text{k}}} = 0.85\,rad} \right)$ of a sample of a native histological biopsy section of a poorly differentiated (4 + 4 Gleason score) carcinoma during radical prostatectomy.

Figures 4 and 5 present exemplar depolarisation maps obtained for samples of prostate adenoma (see Fig. 4), and carcinoma with a 3 + 3 Gleason score (see Fig. 5). However, no obvious differences or relation to the tissue structure are immediately visible. For carcinoma with a higher (4 + 4) Gleason score, more obvious differences are visible. By analysing the results obtained, we can see that there is a complex and individual topographic structure of the depolarisation maps $\Lambda \left( {x,y,\theta_{k} = 0.85\,rad} \right)$ of native histological sections of prostate tumours obtained during radical prostatectomy (Fig. 6).

We can then assess the depolarising ability of histological biopsy sections of benign and malignant prostate tumours within the framework of a statistical approach (15–18) to the analysis of the distributions $\Lambda \left( {x,y,\theta_{k} } \right)$. The largest mean and maximum range of coordinate fluctuations of the magnitude of the degree of depolarisation $\Lambda \left( {x,y,\theta_{k} = 0.85\,rad} \right)$ are found for histological sections of benign adenoma biopsies. The smallest mean and minimum range of coordinate fluctuations of the magnitude of the degree of depolarisation $\Lambda \left( {x,y,\theta_{k} = 0.85\ rad} \right)$ are found for histological sections of poorly differentiated carcinoma biopsies. The experimentally revealed differences in the series of maps $\Lambda \left( {x,y} \right)$ in the phase section $\theta^{*} = 0.85\ rad$ can be associated with the different degrees of differentiation of the structures within the prostate tumour tissues. For well differentiated adenoma tissue, diffraction effects are most pronounced. Therefore, the average value and fluctuations of the degree of depolarisation are maximal. For poorly differentiated (4 + 4) carcinoma tissue, the diffraction broadening of laser waves is smaller. Therefore, both the mean and variance of the individual coordinate values $\Lambda \left( {x,y,\theta_{k} = 0.85\,rad} \right)$ decrease.

The results of quantitative statistical analysis and comparison of the distributions $\Lambda \left( {x,y,\theta_{k} } \right)$ for the different groups of tissues are presented in Table 2. The data obtained shows that statistical moments of higher orders, which characterize the skewness ($Z_{3} \left( {\theta_{k} } \right)$) and kurtosis ($Z_{4} \left( {\theta_{k} } \right)$) of the distributions of the degree of depolarisation, are the most sensitive to changes in the structure of the depolarisation maps $\Lambda \left( {x,y,\theta_{k} } \right)$. Moreover, the maximum differences between the third and fourth order statistical moments ($\Delta \mathop Z\limits_{i = 3;4} \to {\text{max}}$) are realised in the phase section $\theta^{*} = 0.85\,{\text{max}}$. In general, with increasing depolarisation the statistical moments can be characterised by $${{\varvec{\Lambda}}}\left( {{\text{x,y,}}\uptheta_{{\mathbf{k}}} } \right) \uparrow { }\left\{ {\begin{array}{*{20}c} {{\text{Z}}_{{1;2}} \left( {{\Lambda }\left( {{\text{x,y, }}\uptheta_{{\text{k}}} } \right)} \right) \uparrow } \\ {{\text{Z}}_{{3;4}} \left( {{\Lambda }\left( {{\text{x,y, }}\uptheta_{{\text{k}}} } \right)} \right) \downarrow } \\ \end{array} } \right.$$.

**Table 2:**
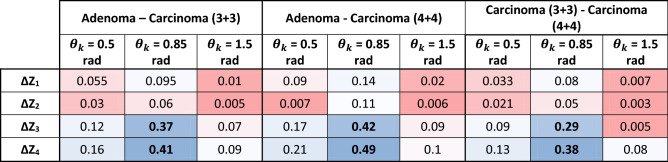
Intergroup differences in the magnitude of the central statistical moments of the 1st–4th orders $\Delta \overline{Z}_{i = 1;2;3;4}$, which characterize the distributions of the magnitude of the degree of depolarisation $\Lambda \left( {x,y,\theta_{k} } \right)$ in the layered phase sections corresponding to $\theta_{k} = 0.5\,{\text{rad}}$, $\theta_{k} = 0.85\,{\text{rad}}$, and $\theta_{k} = 1.5\,{\text{rad}}$.

Table 3 shows the changes in the magnitude of the sensitivity $Se$, specificity $Sp$, and balanced accuracy $Ac$ of the diagnostic power of the 3D Mueller matrix mapping method for native histological sections of benign and malignant prostate tumours.

**Table 3:**
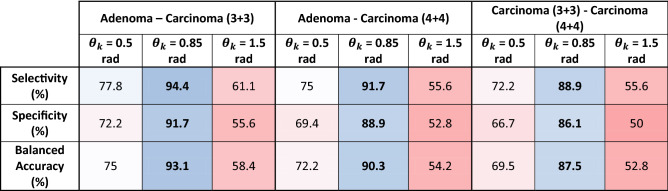
Selectivity, specificity, and balanced accuracy of the diagnostic power of the 3D Mueller matrix mapping method of native histological sections of benign and malignant prostate tumours in the layered phase sections corresponding to $\theta_{k} = 0.5 \,{\text{rad}}$, $\theta_{k} = 0.85\,{\text{rad}}$, and $\theta_{k} = 1.5\,{\text{rad}}$.

In the region of small ($\theta_{k} \le 0.5\,{\text{rad}}$) and large ($\theta_{k} \ge 1.5\,{\text{rad}}$) phase shifts, the diagnostic efficiency of the method is rather low -$69.5\% \le {\text{Ac}}_{{12;13;23}} \left( {\theta_{k} \le 0.5\,{\text{rad}}} \right) \le 75\%$ and $52.8\% \le {\text{Ac}}_{{12;13;23}} \left( {\theta_{k} \ge 1.5\,{\text{rad}}} \right) \le 58.4\%$. However, in the diagnostically optimal ($\Delta Z_{i = 3;4} \to max$) phase section $\theta^{*} = 0.85\ rad$, a high efficiency was revealed not only for differential diagnosis of benign and malignant prostate tumours ($90.3\% \le {\text{Ac}}_{{12;13}} \left( {\theta^{*} = 0.85\,{\text{rad}}} \right) \le 93.1\%$), but also for predicting the severity of the oncological process (${\text{Ac}}_{{{23}}} \left( {\theta^{*} = 0.85\,{\text{rad}}} \right) = 87.5\%$).

## Conclusions

The method of 3D Mueller matrix mapping of diffuse biological layers with digital holographic reconstruction of layered depolarisation maps (1–18) is analytically substantiated. This method, built on the platform of polarisation interferometry, was experimentally tested for the task of express (less than 15 min) differential diagnosis of prostate tumours obtained during radical prostatectomy. A representative sample of diffuse samples of and variously differentiated (Gleason scores 3 + 3 and 4 + 4) carcinoma biopsies was studied. Using the method of phase scanning of the object field of histological sections, the optimal cross section ($\theta_{k} = 0.85\,{\text{rad}}$) was determined. In this cross-section, the maximum differences ($\Delta Z_{{i = {1;2;}3;4}} \to max$) between the values ​​of the central statistical moments $Z_{{i = {1;2;}3;4}}$, which characterize the depolarisation maps of samples of benign and malignant prostate tumours, were realized. The most sensitive statistical parameters were established to be the skewness and kurtosis of the coordinate distributions $\Lambda \left( {x,y,\theta_{k} } \right)$. The operational characteristics (sensitivity $Se$, specificity $Sp$ and balanced accuracy $Ac$) have been determined, which demonstrate the diagnostic power of the 3D Mueller matrix mapping of diffuse biological layers with digital holographic reconstruction of layered depolarisation maps. High accuracy ($90.3\% \le {\text{Ac}}_{{12;13}} \left( {\theta^{*} = 0.85\,{\text{rad}}} \right) \le 93.1\%$) of differentiation of benign and malignant samples of native histological sections of biopsy of prostate tumours was achieved. In addition, the possibility of diagnosing samples of variously differentiated malignant tumours with accuracy ${\text{Ac}}_{{{23}}} \left( {\theta^{*} = 0.85\,{\text{rad}}} \right) = 87.5\%$ has been demonstrated.
